# Cementless unicompartmental knee arthroplasty is safe and effective at a minimum follow‐up of 4.2 years: A systematic review

**DOI:** 10.1002/jeo2.70253

**Published:** 2025-05-07

**Authors:** Pierangelo Za, Giuseppe Francesco Papalia, Umberto Cardile, Pietro Gregori, Sebastiano Vasta, Edoardo Franceschetti, Stefano Campi, Rocco Papalia

**Affiliations:** ^1^ Department of Orthopaedic and Trauma Surgery Università Campus Bio‐Medico di Roma Roma Italy; ^2^ Research Unit of Orthopaedic and Trauma Surgery Fondazione Policlinico Universitario Campus Bio‐Medico Roma Italy; ^3^ Oncological Orthopaedics Department IFO – IRCCS Regina Elena National Cancer Institute Rome Italy

**Keywords:** cemented knee replacements, cementless knee replacements, clinical outcomes, revisions, survivorship, unicompartmental knee arthroplasty

## Abstract

**Purpose:**

Cemented unicompartmental knee arthroplasty (UKA) is a widely used procedure in the treatment of anteromedial and lateral knee osteoarthritis. However, several advantages are reported for cementless UKA, such as improved osseointegration, reduced cement‐related costs and complications. The aim of this study was to analyse clinical outcomes, survival, complications, failures and revision rate of cementless UKA.

**Methods:**

A systematic review was performed on 31 May 2024, on PubMed, Cochrane Library and Scopus. We included randomised clinical trials and prospective and retrospective studies reporting clinical outcomes, implant survival, complications, failures and revision rates of cementless UKA. The following data were extracted: study design, type of implant, number of patients and knees, follow‐up, age, sex, pre‐operative and post‐operative clinical outcomes, reoperations and revisions with causes of failure and overall survival.

**Results:**

Fifteen studies were included, involving 3475 patients and 3641 UKA (2568 cementless UKA and 854 cemented UKA). The mean patients' age was 66 years. The mean follow‐up was 6.5 years. The Oxford Knee Score improved from 17.8 preoperatively to 40.3 post‐operatively in cementless UKA. Knee Society Score improved from 118.2 preoperatively to 168.6 post‐operatively in cementless UKA. The reoperation rate was 3.85% for cementless UKA and 9% for cemented UKA. The most common causes of revision were osteoarthritis progression (1.4%), aseptic loosening (0.8%), bearing dislocation and unexplained pain (0.7%). The overall survival of cementless UKA was 96.2% and 93.6% at 5 and 10 years, respectively.

**Conclusion:**

Cementless UKA is a viable alternative to cemented UKAs with a low failure rate, without a lower clinical benefit at a minimum follow‐up of 4.2 years.

**Level of Evidence:**

Level III, systematic review of studies.

**Abbreviations**
BMI: body mass index
fbUKA: fixed bearing UKA
HA: hydroxyapatite
KSS: Knee Society Score
LOE: level of evidence
mbUKA: mobile bearing UKA
MINORS: methodological index for non‐randomised studies
OA: osteoarthritis
OKS: Oxford Knee Score
PRISMA: Preferred Reporting Items for Systematic Reviews and Meta‐Analyses
PS: prospective studies
RCT: randomised clinical trial
RS: retrospective studies
TKA: total knee arthroplasty
UKA: unicompartmental knee arthroplasty

## INTRODUCTION

Unicompartmental knee arthroplasty (UKA) is a well‐established treatment for isolated end‐stage anteromedial or lateral osteoarthritis of the knee. Cemented fixation is the standard and most diffuse technique for implantation of UKA. However, cementless fixation could be a viable alternative [36]. This option was introduced over 25 years ago with controversial initial results [30, 31, 34, 55]. The early failures of the first uncemented UKA specimens were primarily associated with the properties of the prosthetic materials, particularly the early wear of first‐generation polyethylenes, additionally, aseptic loosening was attributed mainly to polyethylene wear and, to a lesser extent, to inadequate bone‐prosthesis interface surfaces, which compromised proper osseointegration. More recent studies have reported excellent outcomes of cementless UKA [33, 36, 50, 56] largely attributed to the development of advanced materials, including cobalt‐chromium (CoCr), known for its high wear resistance, and oxidised zirconium (Oxinium™), a ceramicized material that reduces wear compared to traditional metals; additionally, bone‐prosthesis interface coatings, such as porous titanium and hydroxyapatite (HA), enhance osseointegration and bone adhesion; furthermore, next‐generation highly cross‐linked polyethylene, optionally supplemented with antioxidants such as vitamin E, has been introduced to minimise wear and extend implant longevity. However, cemented fixation is associated with potential complications such as intraarticular loose cement fragments, excess of cement with impingement and irritation of soft tissues, suboptimal fixation and radiolucent lines (RLs). Conversely, the advantages of cementless UKA include shorter surgical time, reliable fixation, lower incidence of RLs and the avoidance of technical errors in cementation [14, 20, 26, 29, 44, 45]. The introduction of biological fixation could potentially improve implant survival, which is very relevant since UKA is usually performed in younger and more active patients with a longer life expectancy [7, 24]. A systematic review of the most current evidence on cementless UKA was performed with emphasis on clinical outcomes, survivorship, complications, failures and revision rates in order to update existing evidence. The aim of the study was to determine the safety and efficacy of cementless fixation of UKA and whether cementless UKA offers superior mid‐ to long‐term survival while maintaining comparable clinical outcomes to cemented UKA.

## MATERIALS AND METHODS

A systematic review of the literature was performed by two independent reviewers (PZ and GFP) on 31 May 2024 in line with the Preferred Reporting Items for Systematic Reviews and Meta‐Analyses (PRISMA) 2020 guidelines [37]. The following databases were comprehensively searched: PubMed, Cochrane Library and Scopus. The following search string was used: ‘unicompartmental’[All Fields] AND (‘knee’[MeSH Terms] OR ‘knee’[All Fields] OR ‘knee joint’[MeSH Terms] OR (‘knee’[All Fields] AND ‘joint’[All Fields]) OR ‘knee joint’[All Fields]) AND (‘uncemented’[All Fields] OR ‘cementless’[All Fields]). The search process was supervised by a third reviewer (SC). Randomised clinical trials (RCT), prospective or retrospective studies written in English and reporting patient‐reported clinical outcomes, survivorship, complications, failures or revision rates of cementless UKA were included. Both non‐comparative and comparative studies between cementless and cemented were included. Review articles, registers, studies not written in English, case reports, abstracts, hybrid UKRs, all polyethylene tibial components, bicompartmental replacements, studies in which the results of cemented and uncemented UKAs were mixed and studies with follow‐up of less than 4 years were excluded. Titles and abstracts extracted using the previously reported search string were independently examined by two reviewers (PZ and GFP). Abstracts of studies that met the inclusion criteria were examined, and the full manuscripts of the most relevant studies were reviewed to confirm that they met the inclusion criteria. From this list, studies eligible for inclusion were definitively selected by a third examiner (SC). In order to minimise the risk of excluding potentially eligible studies, the references of included studies were also screened. The level of evidence (LOE) of included manuscripts was assessed for therapeutic studies [61]. Subsequently, the following data were extracted: year of publication, study design, LOE, type of implant (cementless or cemented, mobile bearing [MB] or fixed bearing [FB]), number of patients with number of knees, follow‐up, age, sex, body mass index (BMI), preoperative and post‐operative Oxford Knee Score (OKS) and Knee Society Score (KSS), reoperations and revisions with causes of failure, overall survival. The extracted data were organised into tables using Microsoft Excel. In cases of disagreement, data extraction was performed with the consensus of the reviewers (PZ and GFP) and under the supervision of a third reviewer (SC). Risk of bias was independently assessed by two authors (PZ and GFP) using the methodological index for non‐randomised studies (MINORS) criteria.

## RESULTS

### Literature search

**Figure 1:**
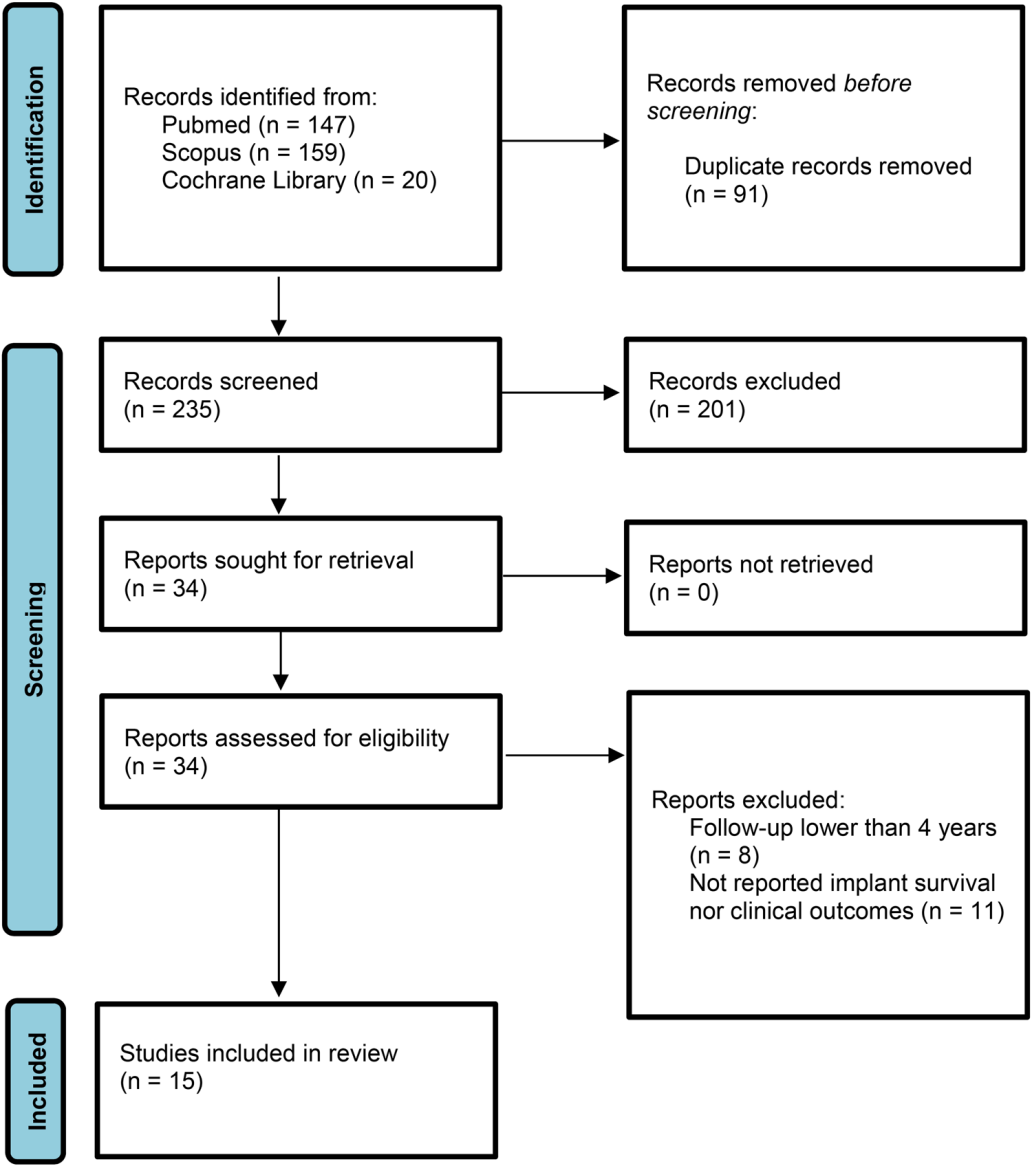
PRISMA 2020 flow diagram. PRISMA, Preferred Reporting Items for Systematic Reviews and Meta‐Analyses.

A total of 326 studies were identified. After the removal of duplicates, 235 articles were selected based on title and abstract. Thirty‐four studies met the inclusion criteria. After examining the full texts, eight articles were excluded due to a follow‐up of less than 4 years and 11 studies were excluded because they did not report clinical outcomes or survival rates. Finally, 15 studies were included, of which 2 were RCT, 4 PS and 9 RS (Figure 1).

### Demographic characteristics

The included studies involved 3475 patients, for 3641 UKA (2568 cementless UKA and 854 cemented UKA). The mean follow‐up was 6.5 years, ranging from 4.2 to 11 years. The mean patients' age was 66 years, ranging from 62 to 72 years. BMI was reported in six studies, and the mean value was 29. A total of seven studies involved both cementless and cemented UKA, while eight studies evaluated cementless UKA only (Table 1).

**Table 1:** Demographic characteristics and clinical outcomes.

Study (year)	Study design	LOE	Implant	Patients (*n*)	Cases (*n*)	Follow‐up (years)	Age	Gender	BMI	Preoperative OKS ± SD	Post‐operative OKS ± SD	Preoperative KSS ± SD	Post‐operative KSS ± SD
Blaney et al. [5] (2017)	RCS	III	Cementless OUKA	238	257	5	65	52%M/48%F	30	16	37	N.R.	N.R.
Kerens et al. [23] (2016)	RCS	III	Cementless OUKA	119	60	4.5	62	52%M/48%F	29	N.R.	39	N.R.	76
			Cemented OUKA		61		63		30	N.R.	39	N.R.	76
Campi et al. [6] (2018)	PCS	II	Cementless OUKA	1120	598	5.3	65	51%M/49%F	N.R.	22 ± 8.1	40 ± 7.9	N.R.	N.R.
			Cemented OUKA		522								
Horsager et al. [15] (2018)	RCT	I	Cementless OUKA	80	25	5	65	60%M/40%F	N.R.	23	39	N.R.	N.R.
			Cemented OUKA		55		63			26	38		
Alvand et al. [1] (2021)	RCS	III	Microplasty OUKA	273	153	6	68	51%M/49%F	N.R.	18	41.5	N.R.	N.R.
			Phase II OUKA		120					19	39		
Nandra et al. [40] (2021)	RCS	III	Cementless OUKA	257	289	5	66	58%M/42%F	N.R.	N.R.	40.1 ± 8.4	N.R.	N.R.
Hall et al. [13] (2013)	RCS	III	Unix cementless	76	85	10	65	57%M/43%F	N.R.	N.R.	38	N.R.	N.R.
Hooper et al. [14] (2015)	PCS	II	Cementless OUKA	126	150	5	64	64%M/36%F	N.R.	22.9 ± 8.4	42.4 ± 6.5	N.R.	N.R.
Jeer et al. [16] (2004)	RCS	III	LCS UKA system	52	66	5.9	69	50%M/50%F	N.R.	20.5	37	N.R.	N.R.
Pandit et al. [45] (2013)	RCT	I	Cementless OUKA	63	30	5	64	53%M/47%	28	21.1 ± 6.1	39.4 ± 9.9	Knee.: 41.6 ± 11.1 Func.: 60.3 ± 13.8 Total: 101.9 ± 17.7	Knee: 78.8 ± 14 Func.: 92 ± 12.7 Total: 170.8 ± 26.7
			Cemented OUKA		33		65	63%M/37.5%	29	21.7 ± 6.4	39.0 ± 10.4	Knee: 44.2 ± 17.7 Func.: 60.6 ± 12.6 Total: 104.8 ± 30.3	Knee: 80.1 ± 19.3 Func.: 78.8 ± 18.4 Total: 158.9 ± 37.7
Lecuire et al. [25] (2014)	RCS	III	Alpina	64	65	11	72	28%M/72%F	28	N.R.	N.R.	Total: 119.3 ± 16.8	Total: 171.4 ± 25.3
Frederix et al. [51] (2022)	PCS	II	Cementless	93 (81 medial and 12 lateral); 68 OUKA, 13 Zimmer and 12 Hermes	28	4.2	N.R.	26%M/74%F	N.R.	N.R.	42	N.R.	N.R.
			Cemented		65								
Kagan et al. [19] (2020)	RCS	III	Zimmer cementless	160 (130 medial and 30 lateral)	41	10	63	63% M/37% F	29	N.R.	N.R.	N.R.	N.R.
			Zimmer cemented		119		70	34% M/66% F	28				
Pandit et al. [46] (2015)	RCS	III	Cementless OUKA	520	579	5.9	65	57%M/43%F	N.R.	27 ± 9	43 ± 7	Knee: 52 ± 20 Func.: 71 ± 17 Total: 123 ± 37	Knee: 81 ± 13 Func.: 86 ± 16 Total: 167 ± 29
Schlueter‐Brust et al. [53] (2014)	PCS	II	AMC/uniglide cementless	234	78	10	69	33%M/67%F	29	N.R.	N.R.	Knee: 33.4 ± 15.9 Func.: 54.7 ± 16 Total: 88.1 ± 31.9	Knee: 94 ± 8.8 Func.: 83.4 ± 17.4 Total: 177.4 ± 26.2
			AMC/uniglide cemented		152								
			Hybrid		10								

Abbreviations: BMI, body mass index; F, female; Func., function; KSS, knee society score; LCS, low contact stress; LOE, level of evidence; M, male; OKS, Oxford Knee Score; OUKA, Oxford unicompartmental knee arthroplasty; PCS, prospective cohort study; RCS, retrospective cohort study; RCT, randomised controlled trial; SD, standard deviation.

MB UKA was used in 11 studies, FB UKA in 3 studies, and both in 1 study. Only medial UKA was performed in 9 studies, while in the remaining 6 studies, both lateral and medial UKAs were analysed.

### Clinical outcomes

OKS was assessed in 12 studies. For cementless UKA, the OKS improved from a mean preoperative value of 17.8 to a post‐operative mean value of 40.3. An excellent score (OKS > 41) was reported in four studies, while the other eight reported a good outcome (OKS 34–41). MB cementless UKA showed a mean post‐operative OKS of 39.7. Focusing on the studies that analysed only medial cementless UKA, the mean post‐operative score was 40. Three studies compared post‐operative OKS between cementless and cemented UKA, and they did not reveal any statistically significant differences.

Moreover, KSS was evaluated in five studies. In cementless UKA, KSS improved from a mean preoperative value of 114.7 to a post‐operative mean value of 169.7. MB cementless UKA reported a mean post‐operative KSS of 147.8. Only one study analysed cemented UKA alone, reporting an increase from 104.8 preoperatively to 158.9 post‐operatively. Combined knee and function scores were reported in four studies, all of which showed excellent outcomes (163–200 points) (Table 1).

### Failures and revisions

All the included studies reported the reoperation rate and the causes of failure. Analysing overall implants, a total of 193 revisions were observed on 3641 cases (reoperation rate 5.3%), with a revision rate of 0.815 per 100 observed component‐years. With cementless UKA, 99 revisions were shown on 2568 cases (reoperation rate 3.85%), with a revision rate of 0.58 per 100 observed component‐years. In the cemented UKA group, there were 77 revisions on 854 cases (reoperation rate 9%), with a revision rate of 1.1 per 100 observed component‐years.

The overall mean revision time after primary UKA was 2.9 years. Moreover, the mean revision time was 3.45 years after cementless UKA and 1.9 years after cemented UKA (Table 2).

**Table 2:** Failures and revisions.

Study	Implant	Mobile bearing (MB)/fixed bearing (FB)	Cases (*n*)	Mean follow‐up in years (range)	Revision	Causes of failure, reoperation (time after primary UKA)
Blaney et al. [5] (2017)	Cementless OUKA	MB	257	5	7	1 Early wound infection (0.5 months) 1 Patient twisted their knee, which resulted in a Grade 2 MCL injury and a bearing dislocation (31 months) 2 OA progression to lateral compartment (45 and 66 months) 1 Injury to knee (69 months) 1 Lateral joint line pain (65 months) 1 Infection (63 months)
Kerens et al. [23] (2016)	Cementless OUKA	MB	60	4.5	5	1 Periprosthetic tibial fracture (1 month) 1 Tibial loosening (2 months) 2 Unexplained pain (18 and 24 months) 1 Synovitis due to RA (21 months)
	Cemented OUKA		61		14	1 Periprosthetic tibial fracture (2 months) 1 Lateral meniscectomy (11 months) 1 Femoral malpositioning (11 months) 5 Unexplained pain (13, 18, 24, 36 and 36 months) 2 Progression of osteoarthritis (20 and 31 months) 1 Femoral loosening (24 months) 1 Infection (36 months) 1 Tibial loosening (36 months) 1 Bearing dislocation (36 months)
Campi et al. [6] (2018)	Cementless OUKA	MB	598	5.3	19	Subsidence (5 months) Synovitis secondary to inflammatory OA (8 months) Avascular necrosis lateral (14 months) Bearing subluxation and anterior impingement (30 months) Unexplained pain (39 months) Progression of osteoarthritis Bearing dislocation
	Cemented OUKA		522		40	Avascular necrosis (9 months) Synovitis and rheumatoid arthritis (25 months) Traumatic periprosthetic femoral fracture (29 months) Subsidence (29 months) Trauma and lateral compartment progression (104 months) Progression of osteoarthritis Bearing dislocation
Horsager et al. [15] (2018)	Cementless OUKA	MB	25	5	2	1 Revision due to bearing dislocation 1 Revision due to loosening
	Cemented OUKA		55			
Alvand et al. [1] (2021)	OUKA	MB	Microplasty 153 Phase III 120	6	Microplasty 3 Phase III 2	4 Lateral progression of OA 1 Prosthetic joint infection (30 months)
Nandra et al. [40] (2021)	Cementless OUKA	MB	289	5	6	1 Lateral compartment symptoms 5 Polyethylene dislocations
Hall et al. [13] (2013)	Unix	FB	85	10	7	4 Aseptic loosening (tibial component), 4 TKA 1 Infection, TKA 2 OA progression, 2 TKA
Hooper et al. [14] (2015)	Cementless OUKA	MB	147	5	6	1 Early loosening of tibia, TKA (1 year) 1 Lateral and PFJ OA progression, TKA (8 years) 2 Bearing dislocations, 1 bearing exchange (1.3 years), 1 ACLR + bearing exchange 2 Late onset of RA, 2 lateral UKAs (4 years)
Jeer et al. [16] (2004)	LCS UKA system	MB	66	5.9	5	2 Lateral OA progression (overcorrection), 2 TKA (5.3 and 5.4 years) 1 Tibial plateau fracture, TKA (2 weeks) 2 Unexplained pain, 2 TKA (0.9 and 1.9 years)
Pandit et al. [45] (2013)	Cementless OUKA	MB	30	5	0	0
Lecuire et al. [25] (2014)	Alpina	FB	65	11	11	2 Lateral OA progression, 2 TKA (1 year, 7 years) 1 Unexplained pain, TKA (8 years) 1 ACL tear, TKA (9 years) 3 PE fractures, 3 revision UKA (4 and 5 years) 4 PE wear, 4 PE exchange (2–6 years)
Frederix et al. [51] (2022)	Cementless/cemented OUKA (*n* 68), Zimmer (*n* 13), Hermes (*n* 12)	MB/FB	81 (mUKA) 12 (lUKA) Cementless 28 Cemented 65	4.2	28	3 Early aseptic loosening (TKA) 1 Symptomatic lateral arthrosis (TKA) 1 Iatrogenic fracture of the tibia (screw osteosynthesis) 2 Stiffness (mobilisation) 6 Prolonged pain 1 Lateral osteoarthritis progression (Osteotomy) 2 Early infection 8 Radiolucent line 4 Technical error in the placement
Kagan et al. [19] (2020)	Cementless/cemented	FB	130 Medial/30 lateral	10	Cemented (15) cementless (8) [medial 21/Lateral 2]	**Cementless** 3 Aseptic loosening 2 Osteoarthritis progression 1 Unknown
						**Cemented** 7 Aseptic loosening 3 Osteoarthritis progression 3 Periprosthetic fracture 1 Infection 2 Unknown
Pandit et al. [46] (2015)	OUKA	MB	579	5.9	6	2 Bearing dislocation (1.8 and 2.3 years) 1 PFJ OA (2.1 years) 2 Progression of OA in lateral compartment (4.2; 6.9 years) 1 Progression of OA in lateral compartment and PFJ (4.0 years)
Schlueter‐Brust et al. [53] (2014)	AMC/uniglide cementless	MB	78	10	2	1 Pain (2.75 years) 1 Bearing dislocation (6.47 years)
	AMC/uniglide cemented		152		7	1 Pain/instability (1.15 years) 1 Pain/stiffness (0.81 years) 1 Pain/synovitis/effusion (4.71 years) 1 Pain/effusion (7.53 years) 3 Bearing dislocation (9.09, 0.20 and 9.70 years)
	Hybrid		10		1	1 Pain/effusion (1.09 years)

Abbreviations: ACLR, anterior cruciate ligament reconstruction; lUKA, lateral UKA; MCL, medial collateral ligament; mUAK, medial UKA; OA, osteoarthritis; OUKA, Oxford unicompartmental knee arthroplasty; PE, polyethylene; PJF, patellofemoral joint; RA, rheumatoid arthritis; TKA, total knee arthroplasty.

The most common causes of revision were osteoarthritis (OA) progression in 52 cases (1.4%), aseptic loosening in 28 cases (0.8%), bearing dislocation and unexplained pain in 25 and 24 cases (0.7%). Finally, 13 failures were due to tibial plateau fracture (0.4%), 8 to infection (0.2%), and 4 to polyethylene wear (0,1%). (Table 3).

**Table 3:** Causes of revision.

Cause	Incidence	Incidence rate (%)
OA progression	52	1.4
Other	39	1.1
Loosening	28	0.8
Bearing dislocations	25	0.7
Unexplained pain	24	0.7
Tibial plateau fracture	13	0.4
Wear	4	0.1
Infection	8	0.2

Abbreviation: OA, osteoarthritis.

### Overall survival

Overall survival was reported in 12 studies. The 5‐year survival of medial MB cementless UKA was reported in nine studies, and the mean value was 96.2%, ranging from 89.7% to 100%. Moreover, the 10‐year survival was reported in four studies, and the mean value was 93.6%, ranging from 91% to 97.4%. Finally, one study reported the overall survival of the Alpina at 13 years, showing a rate of 88%. (Table 4).

**Table 4:** Overall survival.

Study	Implant	Mobile bearing (MB)/fixed bearing (FB)	Medial/lateral	Cases (*n*)	Mean follow‐up (years)	Overall survival
Blaney et al. [5] (2017)	Cementless OUKA	MB	Medial UKA	257	5	96.8% at 5 years
Kerens et al. [23] (2016)	Cementless OUKA	MB	Medial UKA	60	4.5	90% at 34 months
	Cemented OUKA			61		84% at 54 months
Campi et al. [6] (2018)	Cementless OUKA	MB	N.R.	598	5.3	95.8% at 5 years, 91% at 10 years (cumulative)
	Cemented OUKA			522		
Alvand et al. [1] (2021)	OUKA	MB	Medial UKA	153 Microplasty, 120 Phase III	6	99.3% at 5 years
Nandra et al. [40] (2021)	Cementless OUKA	MB	Medial UKA	289	5	97.8% at 5 years
Hall et al. [13] (2013)	Unix	FB	N.R.	85	10	92% at 10 years 76% at 12 years
Hooper et al. [14] (2015)	Cementless OUKA	MB	Medial UKA	147	5	98.7% at 5 years
Jeer et al. [16] (2004)	LCS UKA system	MB	Mixed	66	5.9	89.7% at 5 years
Pandit et al. [45] (2013)	Cementless OUKA	MB	Medial UKA	30	5	100% at 5 years
Lecuire et al. [25] (2014)	Alpina	FB	Mixed	65	11	88% at 13 years
Pandit et al. [46] (2015)	Cementless OUKA	MB	Medial UKA	579	5.9	94.2% at 9 years
Schlueter‐Brust et al. [53] (2014)	AMC/uniglide cementless	MB	Medial UKA	78	10	97.4% at 10 years
	AMC/uniglide cemented			152		95.4% at 10 years

Abbreviations: LCS, low contact stress; OUKA, Oxford unicompartmental knee arthroplasty; SD, standard deviation; UKA, unicompartmental knee arthroplasty.

The area of results reported in each study is shown in Table 5.

**Table 5:** Heatmap showing analysed outcomes. Blue areas represent reported results.

Author	Year	Outcome	OKS	KSS	Revision	Cause of failure	Survivor
Jeer et al. [16]	2004						
Hall et al. [13]	2012						
Pandit et al. [45]	2013						
Lecuire et al. [25]	2013						
Schlueter‐Brust et al. [53]	2014						
Pandit et al. [46]	2015						
Hooper et al. [14]	2015						
Kerens et al. [23]	2016						
Blaney et al. [5]	2017						
Campi et al. [6]	2018						
Horsager et al. [15]	2018						
Kagan et al. [19]	2020						
Nandra et al. [40]	2021						
Alvand et al. [1]	2021						
Frederix et al. [51]	2022						

Abbreviations: KSS, Knee Society Score; OKS, Oxford Knee Score.

### Assessment of risk of bias and publication bias

The included studies were assessed for risk of bias based on the MINORS criteria. Non‐comparative studies achieved an average score of 14.25, whereas comparative studies had a mean score of 20.3 (Table 6).

**Table 6:** Methodological index for non‐randomised studies.

Study	Stated aim	Inclusion of patients	Collection of data	Endpoints appropriate to the aim	Unbiased assessment of study endpoint	Follow‐up	Loss to follow‐up less than 5%	Prospective calculation of study size	Control group	Contemporary groups	Baseline equivalence of groups	Statistical analyses	Total
Blaney et al. [5] (2017)	2	2	2	2	2	2	2	2	NA	NA	NA	NA	16
Kerens et al. [23] (2016)	2	2	2	2	2	1	2	1	2	2	2	2	22
Campi et al. [6 ](2018)	2	2	2	2	0	2	1	1	2	2	2	1	19
Horsager et al. [15] (2018)	2	2	2	2	2	2	1	1	2	2	2	2	22
Alvand et al. [1] (2021)	2	2	2	2	0	2	2	1	2	2	2	1	20
Nandra et al. [40] (2021)	2	2	2	2	0	2	2	2	NA	NA	NA	NA	14
Hall et al. [13] (2013)	2	2	2	2	0	2	2	2	NA	NA	NA	NA	14
Hooper et al. [14] (2015)	2	2	2	2	2	2	1	1	NA	NA	NA	NA	14
Jeer et al. [16] (2004)	2	2	2	2	0	2	0	1	NA	NA	NA	NA	11
Pandit et al. [45] (2013)	2	2	2	2	2	2	2	2	NA	NA	NA	NA	16
Lecuire et al. [25] (2014)	2	2	2	2	0	2	2	2	NA	NA	NA	NA	14
Frederix et al. [51] (2022)	2	2	2	2	0	1	1	0	2	2	2	0	16
Kagan et al. [19] (2020)	2	2	2	2	0	2	1	2	2	2	2	2	21
Pandit et al. [46] (2015)	2	2	2	2	2	2	2	1	NA	NA	NA	NA	15
Schlueter‐Brust et al. [53] (2014)	2	2	2	2	1	2	2	2	2	2	2	1	22

## DISCUSSION

Our main findings were that cementless UKA is safe and effective with good to excellent clinical outcomes and an overall survival of 96.2% and 93.6% at 5 and 10 years, respectively. The initial unsatisfactory results of cementless UKA have been attributed to outdated materials and design [30, 31, 34]. Today, modern implants with cobalt chrome, titanium and an ultra‐high molecular weight polyethylene insert are used and are porous plasma spray and HA coated on all implant/bone interfaces to promote good bone integration. These innovations led to a 10‐year survival rate of 97.4% [53]. Our review reported an overall survival of 96.2% and 93.6% at 5 and 10 years, respectively. This is in line with a previous review reporting an extrapolated 5‐year and 10‐year survival of 96.4% and 92.9%, respectively, for cementless UKA [58]. Mont et al. [38] reported a survival rate of 100% of cementless UKA at a mean follow‐up of 4 years in patients younger than 50 years. Instead, Greco et al. [12] reported a 96% 6‐year survival rate with cemented UKA in patients younger than 50 years. In our review, the median age was 66 years (range, 62–72), and the 5‐year overall survival was 96.2%. Therefore, it is possible that cementless UKA is particularly indicated in young patients. We reported good to excellent clinical outcomes for cementless UKA with respect to KSS (from 118.2 to 168.6) and OKS (from 17.8 to 40.3), which is similar to prior reported clinical outcomes [58]. We also found a lower revision rate for cementless UKA compared to cemented UKA at 3.85% versus 9% respectively. In our review, the most common cause of failure was progression of osteoarthritis, followed by aseptic loosening, which is also reported in prior studies [38, 57]. Similar results were reported for cementless fixed and mobile‐bearing UKA [5]. Aseptic loosening is generally reported as a cause of failure in the early period, while progression of OA occurs more often in mid‐ to late‐term failures [32, 57]. This trend seems to be reversed in the cementless UKA. According to this, we found a mean revision time of 3.45 years for cementless UKA and of 1.9 years for cemented UKA. Campi et al. reported a decrease in aseptic loosening for medial compartment cementless UKA, with the progression of lateral osteoarthritis as the most common cause of failure [6]. This reflects the improved osseous integration and longevity of modern uncemented implants. Anteromedial and lateral knee osteoarthritis can be treated both with UKA and total knee arthroplasty (TKA) [52, 54, 60]. Several retrospective studies analysed cemented fbUKA reporting a survival rate of 98.6% at 2 years [4], 96% at 5 years [59], 92% at 11 years [10] and 83% at 20 years [47]. One randomised controlled trial (RCT) showed a survival rate of 95% at 5 years [9]. These data appear to be comparable to those reported for implants with mbUKA [17]. Equally, there are no differences in clinical outcomes between fbUKA and mbUKA [8, 49]. Data from national registries report a revision rate three times higher for UKA compared to TKA [3, 18, 21, 27, 28, 39, 41, 42, 43, 46]. However, registries do not have information regarding the correct indications of UKA, therefore it is assumed that these data are overestimated. Therefore, a significant reduction in revision rates can be achieved by applying the correct indications and increasing the usage of UKA [21, 22]. All these considerations must also be applied to uncemented UKA. Furthermore, cemented UKAs have a higher radiolucency rate than cementless UKAs [20]. The lower rate of radiolucency reported by cementless UKAs may therefore further reduce the revision rate of UKAs reported by registries. Some considerations must be made regarding the indications when choosing a cementless UKA. Caution should be used in women, especially those over 70 years old [48]. These patients have poor bone quality with a low bone mineral density, which could compromise the integration of the prosthesis and cause early failures [2]. Equal or even better survival was recorded when a cementless arthroplasty was implanted in obese patients [11]. However, these data refer to patients with uncemented TKA and may not be applicable to cementless UKA due to its different mechanical environment at the bone implant interface with lower shared stress, higher compressive loads and less constraint. The results of uncemented UKA have recently been reported in other systematic reviews. In the systematic review by Mohammad et al. [36], 1946 uncemented UKAs were analysed in five studies. In comparison, our systematic review includes 15 studies, with a total of 2568 uncemented UKAs, thus significantly increasing the sample size. This number also exceeds the 1837 cementless UKAs analysed in the review by Mancino et al. [35]. In addition, the mean follow‐up in Mancino's review was 4.1 years, while in our review it extends to 6.5 years, with a maximum follow‐up of 11 years. The larger cohort and extended follow‐up period increase the robustness of our analysis compared to previous reviews, providing a more comprehensive update of existing evidence in the literature.

Our review has several limitations. No specific subgroup analyses were performed, as the heterogeneity among the included studies in terms of implant type, patient characteristics, and reporting methods did not allow for a statistical comparison. Moreover, the studies did not account for potential confounding factors such as age or BMI. Furthermore, some studies report mixed results for both medial and lateral UKA. Therefore, some results are not specific to medial or lateral UKA. Since some differences were found for cemented UKA between the lateral and medial sides, the same differences could be noted between medial and lateral cemented UKA. Further studies are necessary to isolate the two groups. Moreover, our average follow‐up is 6.5 years. Although the use of cementless UKA is safe, further studies are needed to demonstrate its effectiveness in longer follow‐up. Finally, different prosthetic implants are included in our review. While all implants are modern, some designs may be lower performing than others and may therefore negatively influence the results.

## CONCLUSION

Cementless UKA is safe and effective alternative to cemented UKAs with a low failure rate without a lower clinical benefit at a minimum follow‐up of 4.2. Correct indications and proper technique are key to reducing revision rates. Further studies are needed to evaluate its effectiveness in specific populations (women, over 70s, obese) as well as differentiating results for medial and lateral UKAs.

## AUTHOR CONTRIBUTIONS

All authors contributed to the study conception and design. **Pierangelo Za**: Conceptualisation; data curation; formal analysis; writing—original draft. **Giuseppe Francesco Papalia**: Conceptualisation; data curation; formal analysis; writing—original draft. **Stefano Campi**: Conceptualisation; project administration; writing—original draft. **Umberto Cardile**: Data curation; formal analysis; writing—original draft. **Edoardo Franceschetti**: Funding acquisition; project administration; writing—review and editing. **Rocco Papalia**: Funding acquisition; supervision. **Pietro Gregori**: Methodology; writing—review and editing. **Sebastiano Vasta**: Methodology; supervision; writing—review and editing.

## CONFLICT OF INTEREST STATEMENT

The authors declare no conflicts of interest.

## ETHICS STATEMENT

Ethics approval and informed consent were not required for this systematic review.

## Data Availability

Data sharing is not applicable to this article as no data sets were generated or analysed during the current study.
